# Exploring Fixation Times During Emotional Decoding in Intimate Partner Violence Perpetrators: An Eye-Tracking Pilot Study

**DOI:** 10.3390/brainsci15070732

**Published:** 2025-07-08

**Authors:** Carolina Sarrate-Costa, Marisol Lila, Luis Moya-Albiol, Ángel Romero-Martínez

**Affiliations:** 1 Department of Psychobiology, Faculty of Psychology, University of Valencia, 46010 Vealencia, Spain; carolina.sarrate@uv.es (C.S.-C.); luis.moya@uv.es (L.M.-A.); 2 Department of Social Psychology, Faculty of Psychology, University of Valencia, 46010 Vealencia, Spain; marisol.lila@uv.es

**Keywords:** emotion recognition, fixation times, eye tracker, intimate partner violence

## Abstract

Background/Objectives: Deficits in emotion recognition abilities have been described as risk factors for intimate partner violence (IPV) perpetration. However, much of this research is based on self-reports or instruments that present limited psychometric properties. While current scientific literature supports the use of eye tracking to assess cognitive and emotional processes, including emotional decoding abilities, there is a gap in the scientific literature when it comes to measuring these processes in IPV perpetrators using eye tracking in an emotional decoding task. Hence, the aim of this study was to examine the association between fixation times via eye tracking and emotional decoding abilities in IPV perpetrators, controlling for potential confounding variables. Methods: To this end, an emotion recognition task was created using an eye tracker in a group of 52 IPV perpetrators. This task consisted of 20 images with people expressing different emotions. For each picture, the facial region was selected as an area of interest (AOI). The fixation times were added to obtain a total gaze fixation time score. Additionally, an ad hoc emotional decoding multiple-choice test about each picture was developed. These instruments were complemented with other self-reports previously designed to measure emotion decoding abilities. Results: The results showed that the longer the total fixation times on the AOI, the better the emotional decoding abilities in IPV perpetrators. Specifically, fixation times explained 20% of the variance in emotional decoding test scores. Additionally, our ad hoc emotional decoding test was significantly correlated with previously designed emotion recognition tools and showed similar reliability to the eyes test. Conclusions: Overall, this pilot study highlights the importance of including eye movement signals to explore attentional processes involved in emotion recognition abilities in IPV perpetrators. This would allow us to adequately specify the therapeutic needs of IPV perpetrators to improve current interventions.

## 1. Introduction

Intimate Partner Violence (IPV) against women is a major public health and social problem. It involves a wide range of physical, psychological, and sexual abusive behaviors perpetrated by a heterosexual male partner within their intimate relationships [1]. The recurrence and severity of IPV, as well as its psychological impact on victims, highlight the need for suitable interventions [2], although those developed so far report limited effectiveness in reducing recidivism rates [3].

Research has highlighted socio-emotional deficits (such as alterations in emotional decoding, alexithymia, and low empathy) as important factors in IPV perpetration [4,5,6]. In this sense, alterations in identification and communication of emotional states in IPV perpetrators have been linked to dysregulations in autonomic and psychological regulation when dealing with acute stress [7], facilitating, in turn, aggressive behavior [8]. Several authors have demonstrated the suitability of assessing emotion recognition through psychophysiological signals [9,10]. However, as far as we know, there is no research from a biopsychosocial perspective that incorporates psychophysiological markers to assess emotional decoding abilities in IPV perpetrators, which may be crucial to understanding and addressing the risk of violence [11]. Thus, a better understanding of the emotional processing mechanisms could promote the development of targeted, evidence-based interventions aimed at IPV perpetrators.

The advancement of new technologies allows us to investigate cognitive and emotional processes in more detail [12]. In fact, current instruments used to measure emotion recognition often have a limited validity [13]. This limited validity may be due to biases and potential distortions, such as social desirability and lack of honesty [14,15]. This highlights the need to develop or use instruments that can overcome these biases in the reports used to measure these abilities in IPV perpetrators [16,17]. The use of self-reports asking participants to identify emotions from facial pictures has also been reported to have limited reliability [18]. In this regard, integrating neuroscience methods can provide an excellent framework to understand objective and unconscious insights in this population, complementing psychological assessments [14,19]. Among the available physiological instruments, eye tracking has emerged as a valuable tool in neuroscience, offering new possibilities to detect and process emotional responses based on ocular metrics [12,20].

In daily life, we constantly analyze faces to interpret emotions [21]. The ability to correctly interpret others’ emotions through their facial expressions is crucial for effective social and affective interactions [22,23]. Moreover, it serves as a valuable source of information for the assessment of empathic tendencies [24]. That is, this source of information may affect the capacity to understand (cognitive empathy) and/or share the feelings of others (emotional empathy). The Social Information Processing model [25] establishes that individuals with aggressive tendencies often struggle to recognize complex emotions, with a bias towards negative emotional interpretations. These alterations increase their vulnerability towards impulsive and violent responses, especially in conflictive and social situations [25].

In line with the above, deficits in the Social Information Processing model have been related to IPV perpetration [26,27]. Concretely, several studies have concluded that men convicted of IPV tend to present poorer recognition capacities compared to non-violent men or control groups [28,29,30]. While our study does not include a control group to replicate such comparisons, this evidence provides a theoretical basis for exploring the mechanisms underlying emotional decoding within this population. Additionally, worse emotional decoding ability in IPV perpetrators has been associated with higher levels of alexithymia (difficulties in identifying, describing, and expressing emotions) [6,31,32]. Some authors have further concluded that poorer emotional decoding capacities combined with deficits in attention switching and set shifting decrease the ability to recognize IPV in a set of clips showing couples arguing and increase the acceptability of attitudes towards IPV perpetration [33].

Eye tracking is a widely used technique to assess visual attention by measuring where and for how long a person fixates on different elements within a stimulus [34]. Eye trackers provide various measures of visual attention, including point of gaze, fixation duration, saccades, and pupil size, among others [35]. These signals provide objective metrics that can be used to comprehend the way a person is interested in, processes, and respond to the environment [36], reporting data about their visuospatial information processing [37] and even their emotion recognition capacity [38]. Studying eye movement patterns associated with successful emotion recognition provides a reference framework for understanding the diversity of perceptual strategies that may underlie optimal performance [39]. Thus, fixation times in emotional identification help detect which area of the screen captures the user’s attention, through the establishment and analysis of areas of interest (AOI) [38,40].

So far, only a few studies that have used eye tracking during emotion recognition have focused on analyzing fixation times on specific facial regions [40,41]. These studies have found that, depending on the type of emotion, people rely on different facial features to identify it [38,40,41,42]. Additionally, individuals with empathy deficits seem to spend less time analyzing facial features compared to controls [38]. More specifically, it has been shown that reduced and shorter fixation times on the eye region of emotional faces in children and adolescents with psychopathic traits, as well as psychopathic adults [43,44,45], are linked to poorer emotion recognition [43]. In line with these findings, recent research indicates that violent offenders show fewer initial gaze shifts to the eyes, although their total fixation time on this region appears to be preserved, suggesting possible compensatory mechanisms during later processing stages [46]. Despite sufficient evidence that attention to the eye regions (established as AOI) is important for understanding social information processing [44], some authors have discussed the need to include other relevant facial areas (such as the mouth) that could provide significant information, adding ecological validity [47]. Studies analyzing fixation times on the entire face have reported that violent offenders exhibit reduced attention orienting to faces [48]. However, to our knowledge, this is the first study to specifically explore the relationship between total fixation times on the whole face area and emotional decoding abilities in IPV perpetrators.

It is known that cognitive state influences the ability for emotion recognition. Specifically, good performance on tasks assessing executive functions (mental skills that help manage thoughts, actions, and emotions to reach goals such as attention, planning, flexibility, problem-solving…) has been linked to a greater ability to accurately perceive emotional expressions [30,49,50]. In fact, the recognition of emotional facial expressions requires conscious awareness [51], which suggests that attentional control plays a key role in facilitating emotional processing. Conversely, deficits in attention are likely to impair this ability. Impaired executive functions in attention have been found to reduce fixation times, indicating less effective and comprehensive encoding of the visuospatial information in the image [52,53]. Therefore, considering that individuals with IPV exhibit substantial impairments in these cognitive domains [54,55] and that fixation patterns are positively related to cognitive functions [53] and are considered a reflection of attention capability [40], neuropsychological profiles should be kept in mind.

Keeping all this in mind, the main aim of the present study was to examine the association between fixation times from the eye tracking register during an emotion recognition task and emotional decoding abilities in a group of IPV perpetrators, controlling for potential confounding variables. Specifically, cognitive flexibility and attention would be controlled, as previous studies have shown their direct influence on fixation times [40,53]. Several studies have concluded that IPV perpetrators tend to present poorer recognition capacities compared to non-violent men or control groups [28,29,30]. While our study does not include a control group to replicate such comparisons, this evidence provides a theoretical basis for exploring the mechanisms underlying emotional decoding within this population. Based on previous conclusions in this field of research [45,48], it was expected that fixation times would explain a significant percentage of the value observed in our ad hoc emotional decoding test, even when controlling for covariates, with longer fixation times being associated with a greater ability to identify emotions.

## 2. Materials and Methods

### 2.1. Participants

After excluding participants with missing data due to a suboptimal calibration in the eye tracker (n = 1) or unanswered items or incomplete neuropsychological tests (n = 3), the sample was composed of 52 men convicted of IPV. These men had been assigned to a community-based prevention program for IPV (Programa CONTEXTO) as part of a court order. This program is aimed at gender-based violence offenders who have received a prison sentence of less than two years and have no prior criminal records [56].

The inclusion criteria for this study were: (a) to not suffer from any congenital or acquired neurological disorders (e.g., neurodevelopmental or neurodegenerative diseases, traumatic brain injuries, brain tumors…) or mental disorders (schizophrenia, depression, etc.), + assessed using the Symptom Checklist-90 [57], (b) to have a good comprehension of Spanish, and (c) to not suffer from any ocular disease (e.g., strabismus) that would hinder rigorous eye-tracking recording and interfere with fixation times, given how individuals with strabismus exhibit longer fixation times [58].

All participants were informed about the protocol to be followed throughout the research, participated voluntarily, and provided informed consent prior to their participation in the study. Furthermore, it is important to note that this study was conducted in accordance with the principles established in the Declaration of Helsinki and received approval from the Ethics Committee of the University of Valencia (assigned codes: H1515749368278 and 2024-PSILOG-3527732).

### 2.2. Procedure

For the present study, a session lasting approximately an hour and a half was conducted in the laboratories of the Psychobiology Department at the Faculty of Psychology of the University of Valencia. All evaluations took place between 10:00 and 13:00 in the morning to avoid the effects of fatigue caused by the passage of the day.

Participants were taken to a soundproof room to minimize distractions as much as possible. At the beginning of the session, participants signed an informed consent form. Then, a short and structured interview was conducted to collect information on sociodemographic variables. Subsequently, the eye-tracking procedure was carried out, which lasted approximately 15 min. During the image presentation, specifically following each image, the items from the ad hoc emotional decoding test were individually administered. Afterward, information about objective emotional decoding abilities was collected through the Reading the Mind in the Eyes Test (or eyes test) and the Emotional Identification subscale of the Toronto Alexithymia Scale-20 (TAS-20), as a subjective emotional decoding measure. Finally, the Wisconsin Card Sorting Test and the Conners’ Continuous Performance Test-III were administered by trained professionals.

### 2.3. Instruments

#### 2.3.1. Eye Tracker

We evaluated eye movements of the participants using the Tobii Pro Nano 60 Hz (2.5 version) sampling rate eye tracker. This compact, lightweight device connects via USB to a computer and offers a high tolerance for head movements and lighting variability, minimizing data loss due to blinking. The system automatically captures and analyzes eye movements by detecting corneal reflections and pupillary contrast against the white sclera. These movements are recorded as x and y coordinates over time. The device was mounted onto a 16″ screen of a laptop (Lenovo ThinkBook, Morrisville, NC, USA), positioned 65 cm from the participants, and the participants remained seated upright with a stable backrest.

Before starting the task, a rigorous 9-point calibration and validation process was conducted to ensure consistency in eye movement measurements. Only participants who achieved satisfactory calibration according to the manual (typically corresponding to a mean error below ~1° of visual angle) were included in the analysis [59]. After calibration, participants were instructed to pay attention to the images and were informed that, after each image, they would be asked to identify (through a multiple-choice question) the emotion felt by the person or people depicted. The task included 20 images.

The data analysis was performed using Tobii Pro Lab (2024) [60]. We focused our analysis on the fixation time on the AOI. Fixation time was measured in milliseconds within manually defined face regions expressing specific emotions (without other disturbances such as hair, accessories…), which were selected as AOIs. Fixation events were identified using the I-VT (Velocity-Threshold Identification) filter with a threshold of 30°/s and a minimum fixation duration of 60 ms, as implemented in Tobii Pro Lab. We obtained a total fixation time by adding the time spent on each fixation event on the different AOIs. According to previous research, fixation time was considered a valid indicator of visual processing [61,62]. Indeed, this tool has been previously used as a useful method to assess visual attention patterns during emotional decoding tasks [63,64,65].

#### 2.3.2. Visual Stimuli Presentation During the Emotion Recognition Task

A total of 20 photographs were selected from the Pexels website (https://www.pexels.com/es-es/, accessed on 28 June 2022), It is a freely accessible and usable website that allows the use of images for personal, commercial, and academic purposes without the need for attribution. The selection criteria required that each image include at least one visible face displaying a clear or identifiable emotional expression in different contexts. All faces had to be unobstructed (i.e., no sunglasses, masks, or heavy shadows), although in some images only part of the face was visible due to the natural context. Images showing one to three people were included. The final set contained an equal number of positive and negative valence images (50/50). The pictures include IPV (both with male and female offenders) and ordinary situations (e.g., a father playing with his child, a woman handing out a report at her workplace, or an argument between two individuals).

**Figure 1:**
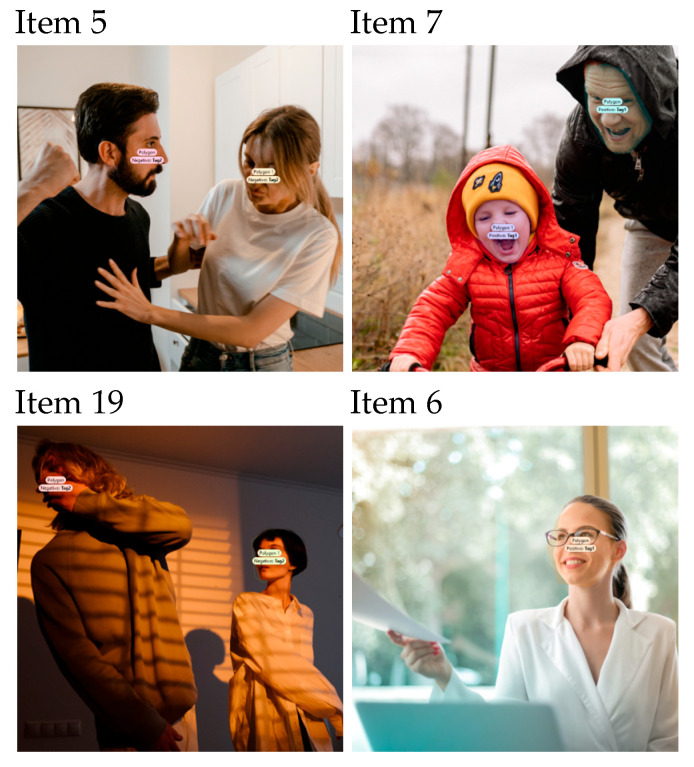
Example items with specified areas of interest.

We offer some examples with their respective AOIs (facial expressions) in the figure below (Figure 1). Each image may include one or multiple AOIs.

To maintain standardized conditions, all participants viewed the same set of images in a controlled environment with uniform lighting and seating arrangements. Each image was shown for a fixed duration of 7 s.

#### 2.3.3. Ad Hoc Emotional Decoding Test

An ad hoc multiple-choice scale was created to assess the emotional decoding abilities of images presented on the computer’s screen during eye-tracking registration. Immediately after each image disappeared from the screen, a black screen appeared, and the evaluator orally asked a closed multiple-choice question regarding the emotional state of the person or people in the image. The participant responded verbally, and the evaluator registered the answer. To ensure standardization across participants, all questions and their four fixed response options were always read aloud in the same order. This procedure minimized variability and ensured consistency in the verbal response format.

The total number of items was 34 (items of each face displayed during the task). Correct answers were established by expert consensus (two psychologists) that independently rated the intended emotional response for each image, and discrepancies were resolved via discussion until unanimous agreement was reached. They were scored with 1, while incorrect answers were scored with 0. The total score on the scale is obtained by adding all correct responses. A higher score indicates a greater capacity for emotion recognition. The Cronbach’s alpha reported for the first version of this scale was 0.54, and the item-level analysis indicated that removing any item did not substantially improve reliability (maximum α = 0.56). Moreover, when splitting the items by emotional valence, internal consistency further decreased (positive valence α = 0.44; negative valence α = 0.32). The reliability value found is similar to those reported by other instruments that assess emotion recognition, such as the Eyes Test. In a recent analysis of the psychometric properties of this test, it was found that its reliability values (based on Cronbach’s alpha) ranged from 0.45 to 0.96 [66]. Through their meta-analysis, these authors estimated that the internal consistency was acceptable (α = 0.73). However, a previous study suggested that the internal consistency of the Eyes Test is modest, with Cronbach’s alpha varying from 0.37 to 0.61 depending on cultural adaptations [18].

#### 2.3.4. Reading the Mind in the Eyes Test (Eyes Test)

To evaluate emotional decoding abilities, we used the Reading the Mind in the Eyes Test, commonly referred to as the Eyes Test [67]. This test comprises 36 black-and-white images displaying the eye region of various men and women. Each image is accompanied by four possible answers that describe emotions, from which participants must choose one. The total score ranges from 0 to 36, with higher scores reflecting greater emotional decoding skills. In this study, Cronbach’s alpha was 0.53. This instrument was originally designed to evaluate the Theory of Mind, and its use to assess emotion recognition has been questioned [18]. However, its relationship with emotion recognition is moderate [66], supporting its application for this purpose. Moreover, most of the studies that assess emotional decoding in IPV perpetrators use this instrument [28,30].

#### 2.3.5. Toronto Alexithymia Scale-20 (TAS-20)

To assess self-reported emotional decoding capability, we used the emotional identification subscale of the Spanish version [68] of the Toronto Alexithymia Scale-20 (TAS-20) [69]. This tool includes 20 items rated on a 6-point Likert scale, ranging from 1 (strongly disagree) to 6 (strongly agree). The emotional identification score is obtained by adding the next item scores (1, 3, 6, 7, 9, 13, 14), with higher scores indicating a lower auto perception of the ability of emotional identification. In this study, Cronbach’s alpha for this subscale was 0.81.

#### 2.3.6. Wisconsin Card Sorting Test (WCST)

We employed the Wisconsin Card Sorting Test (WCST) [70] to evaluate executive functions. The percentage of perseverative responses was used in this study, as it serves as a general indicator of test performance. This score represents the number of times the participant repeats a specific response even after being informed that it is incorrect. It is an indicator of cognitive flexibility and executive control. In this way, a higher number of perseverative responses reflects a lower ability to adapt and follow new rules set by the evaluator (greater mental rigidity) and reduced inhibitory control. Higher scores on the percentage of perseverative responses reflect worse overall performance.

#### 2.3.7. Conners’ Continuous Performance Test-III (CPT-III)

The Conners’ Continuous Performance Test-III (CPT-III) [71] was used to assess attention. The evaluator instructs the participants to press the space bar whenever a letter appears on the screen, except when the letter “X” appears. In this study, we used the score for “number of omissions” as a general indicator of performance on the test. This score represents the number of times the participant fails to press the space bar in response to a non “X” letter. Therefore, a higher score (greater number of omissions) indicates poorer attentional capacity (sustained and selective attention, and vigilance).

### 2.4. Data Analysis

Initially, we provided the descriptive data of the sociodemographic factors (age, nationality, educational level, marital status, and employment status), fixation times, emotional decoding instruments (ad hoc emotional decoding test, the Eyes Test, and TAS-20), and neuropsychological tests (WCST and CPT-III) of the sample.

Afterwards, to analyze the relationship between fixation times and emotion recognition tests, we performed partial correlations between fixation times and the emotional decoding instruments, including the WCST percentage of perseverative responses and CPT-3 omissions as covariates. After confirming a significant correlation between fixation times and the ad hoc emotional decoding test, we conducted a linear regression to thoroughly examine this relationship. Fixation time was treated as the independent variable, and the score obtained in the emotional decoding test was the dependent variable. In step 0, the covariates mentioned above were entered into the model. In step 1, the independent variable was added to assess its incremental predictive value.

All statistical analyses were conducted using IBM SPSS Statistics for Windows, Version 26.0, with the significance level set at 0.05.

## 3. Results

### 3.1. Sample Description

The following section presents the descriptive sociodemographic characteristics, the emotional decoding scores in the different instruments, eye-tracking total fixation times, and neuropsychological scores of the entire sample (Table 1).

### 3.2. Partial Associations Between Fixation Times and Emotional Recognition Tests

As shown in Table 2, a positive correlation was found between fixation times and our ad hoc emotional decoding test, even while controlling for potential confounding variables (neuropsychological tests), indicating that longer visual engagement with emotional faces is related to better recognition of emotions. Moreover, there was a significant correlation between our ad hoc emotional decoding test and the eyes test, as well as between this instrument and the Emotional Identification subscale (EIS) of the TAS-20, suggesting a certain degree of convergence between the different measures of emotional decoding.

### 3.3. Fixation Times for Explaining Emotional Decoding Abilities

Regression analyses revealed that fixation times in the AOI (facial area) significantly explained 20% of the variance in the score in the ad hoc emotional decoding test (Table 3).

In greater detail, in step 0, the model was not statistically significant, although the CPT-III (omission errors) emerged as a significant negative predictor of emotional decoding performance, indicating that poorer attention was associated with lower decoding accuracy. In Step 1, the model showed an improved fit, with a statistically significant increase in explanatory power. Fixation time significantly predicted better emotional decoding performance, suggesting that longer fixation on emotional faces is positively associated with decoding ability, even after controlling for attention and executive functions.

## 4. Discussion

The present study aimed to examine the association between fixation times on facial images and emotional recognition and/or emotional decoding abilities in IPV perpetrators. Our data revealed a significant association between fixation times and emotional decoding abilities, even when controlling for confounding factors. Specifically, fixation times explained 20% of the variance observed in the scores obtained using the ad hoc emotional decoding test. This suggests that fixation time on faces may serve as a relevant attentional marker associated with emotion recognition performance in IPV perpetrators. Therefore, our findings open new avenues for investigating the role of visual attention in socio-emotional functioning within this population, using physiological measures such as eye tracking.

In the first part of the present study, we aimed to explore the relationship between the different variables of interest. As prior research has consistently reported emotional decoding difficulties [28,29,30], our objective was to examine how attentional engagement with emotional facial expressions (through fixation time) is associated with emotion recognition abilities within this specific population. We found that longer fixation times on the AOI (facial expressions) were associated with higher accuracy in the ad hoc emotional decoding test, even after accounting for potential confounding variables. Our instrument also showed significant associations with previous tests, such as the Eyes Test and the emotional identification subscale of the TAS-20, with the expected direction of these associations. Most importantly, the ad hoc recognition instrument demonstrated reliability similar to that of the Eyes Test. Although it was still modest and may reflect some degree of measurement error, this suggests the potential to develop new instruments to complement existing ones for measuring emotion recognition and decoding abilities.

The second part of this study assessed the explanatory effect of fixation times on emotional decoding ability. It was found that longer fixation times on the AOI on static emotional faces explained 20% of the observed variations in emotional decoding scores. The strength of the observed association indicates that fixation times account for a relevant portion of the variance in emotional decoding performance. This supports the relevance of attentional mechanisms in explaining individual differences in emotion recognition skills among IPV perpetrators. Hence, eye tracking could be considered in future experimental protocols as a complementary instrument for a more rigorous assessment of emotional decoding abilities. So far, this instrument and the analysis of eye movements have been validated as systems for recognizing individuals’ emotions, achieving moderate levels of accuracy [72,73]. However, the eye features (such as gaze movements, duration of fixation times…) have not been used or validated to explore emotional decoding abilities when observing others experiencing different emotions.

This study aligns conceptually with the Social Information Processing model by providing complementary support of the attentional mechanisms relevant to the encoding and interpretation of the social information phase, which refers to how individuals attend to and extract information from social stimuli [25]. The observed association between fixation times and emotional decoding abilities in IPV perpetrators may reflect attentional mechanisms relevant to how social cues are processed and interpreted. This provides preliminary support for integrating ocular metrics, such as those derived from eye-tracking, into broader theoretical models of violent behavior. In this way, fixation times may be considered a key factor in understanding information processing that underlies socio-emotional difficulties. This could be explained by the fact that empathy deficits have been partly attributed to impairments in social information processing, such as misunderstanding others’ intentions [74], which in turn, has been linked to poorer anger management [75]. In fact, a hostile interpretation bias toward facial expressions has been linked to antisocial or borderline personality traits, as well as a pathological tendency for aggression [76]. Additionally, emotional decoding deficits have been linked to emotional and behavioral dysregulation [77], which is widely manifested in this sample [78]. Hence, this could increase the vulnerability toward violent behavior, as demonstrated in IPV perpetrators [79,80]. Note that emotion recognition and cognitive empathy were strongly and negatively linked to dropout and recidivism in this group [7,11,80]. Consequently, interventions focused on improving emotional processing and decoding by promoting the maintenance of visual attention on significant social stimuli (such as emotional faces) through biofeedback tasks could enhance emotional regulation in this population.

Given that difficulties in emotionally connecting with others’ negative emotions have been considered a risk factor for IPV perpetration, some authors have supported the inclusion of empathy training in interventions aimed at IPV perpetrators. They hypothesize that interventions focused on enhancing socioaffective functioning would reduce IPV perpetration [5,28]. Interventions carried out with violent offenders show that including emotion recognition training in the programs enhances emotional decoding abilities and reduces the likelihood of violence [81,82]. In the case of IPV perpetrators, it has only been demonstrated that training these skills has a beneficial effect specifically on cognitive functioning when combined with conventional treatments [83]. In contrast, programs that focus on improving empathic capacity have been considered effective in reducing aggression in this group [84]. Therefore, our study contributes to the design of innovative interventions that incorporate visual attentional retraining or social information processing strategies as a pathway to improve emotion recognition and, ultimately, empathy, thereby reducing violent behavior in IPV perpetrators.

This study represents an initial attempt to explore the association between visual fixation (via eye tracking) on the whole face area of expressive faces (during a task designed to represent real-life emotional stimuli) and emotion recognition ability in IPV perpetrators using an ecologically valid task. Thus, it expands upon previous findings and offers a complementary perspective on the attentional processes involved in the assessment of emotional decoding abilities. Nonetheless, our pilot experimental protocol has significant limitations. First, due to the specificity of the sample (IPV perpetrators) and the preliminary nature of the study, it represents an initial exploration with a relatively small sample size, which may affect the statistical power and generalizability of the results. In the future, we aim to recruit more participants and validate our findings in a larger population. Additionally, the study relies on static images of 7-s exposure. Thus, their ecological validity could be limited, as they do not include dynamic cues (such as movement, voice tone…) and natural variability in how people process emotional cues in real-life contexts [13]. Future research should aim to incorporate eye tracking in videos. Moreover, given that some images feature repeated actors while others do not, this could introduce bias, as humans tend to maintain consistent fixation patterns on the same face identity even across different expressions [85]. Furthermore, the emotional decoding task was specifically designed to enhance ecological validity by presenting emotional images in socially meaningful contexts, rather than isolated facial expressions. Despite this effort to represent real-life emotional situations, the cognitive demands inherent to an explicit recognition task may still differ from those involved in spontaneous emotional processing during natural interactions. Moreover, the internal consistency of our ad hoc emotional decoding test was modest (α = 0.54). These values are comparable to those observed in widely used tools in the field, such as the *Reading the Mind in the Eyes Test* [18]. While acknowledging this limitation, we argue that the ecological validity and feasibility of our instrument justify its use in this pilot context. Future work should aim to enhance its psychometric properties. As this was an exploratory pilot study, our primary objective was to test the feasibility of combining a context-rich emotion recognition task with eye-tracking methodology. Future research should aim to improve the psychometric properties of the instrument and examine whether attentional mechanisms differ across spontaneous and non-spontaneous emotion processing tasks. Additionally, the lack of a non-violent control group limits the ability to determine whether the observed association between attention to faces and emotion recognition differs between IPV perpetrators and community samples. For this reason, the findings are exploratory and correlational in nature and should not be interpreted as indicating deficits or atypical functioning in IPV perpetrators. The study is limited to the description of attentional–emotional associations within this population. The decision to focus on this group was based on a substantial body of literature documenting emotion recognition difficulties in IPV perpetrators [28,29,30]. Future research should aim to validate this task in non-violent community samples to establish normative performance ranges, further refine the task’s psychometric properties, and clarify the specificity of these findings. Finally, this study focuses solely on the metric of fixation time on the AOI. However, other eye registrations (point of gaze, saccade movements…) could also provide valuable insights into attentional and processing strategies [39,41]. Thus, combining different eye-tracking features could enhance the accuracy, interpretability, and reliability of the assessments based on eye-tracking in this context.

## 5. Conclusions

In summary, this preliminary study opens new avenues in the research of emotional decoding ability through eye movement analysis. It contributes to the understanding of the attentional mechanisms involved in emotion recognition through eye tracking and ultimately to the design of interventions to improve emotion recognition in IPV perpetrators.

## Figures and Tables

**Table 1:** Participant characteristics (mean, standard deviation, and percentages).

	IPV Perpetrators (n = 52)
Age (M, SD)	42.98 ± 11.76
Nationality (%)	
Spain	76.9
South America	9.6
Eastern Europe	7.7
Africa	5.8
Educational level (%)	
No formal education	9.6
Primary/elementary education	51.9
Secondary school or vocational training	32.7
University education	5.8
Marital status (%)	
Single	55.8
Married	19.2
Divorced	23
Employment status (%)	
Employed	51.9
Unemployed	48.1
Total fixation time (ms)	100,051.32 ± 33,246.80
Ad hoc emotional decoding test	25.52 ± 2.64
Eyes Test	19.88 ± 4.36
TAS-20 (Emotional Identification subscale)	17.68 ± 7.52
WCST (Perseverative responses)	25.25 ± 14.70
CPT-III (Omissions)	2.54 ± 4.20

Note. M: mean; CPT-III: Conners’ Continuous Performance Test-III; IPV: intimate partner violence; SD: standard deviations; ms: milliseconds; TAS-20: Toronto Alexithymia Scale-20; WCST: Wisconsin Card Sorting Test.

**Table 2:** Partial correlations between the study variables.

	Ad hoc Emotional Decoding Test	Eyes Test	TAS-20 (EIS)
Total fixation time (ms)	0.371 *	0.234	−0.137
Ad hoc emotional decoding test		0.291 *	−0.288 *
Eyes Test			−0.157

Note. EIS: Emotional Identification Subscale; ms: milliseconds. * *p* ≤ 0.05.

**Table 3:** Regression analysis between fixation times and ad hoc emotional decoding test.

Emotional Decoding							
Effect	B	95% CI for B		SE B	Β	R^2^	ΔR^2^
		LL	UL				
Step 0							
Constant	26.40	24.70	28.10	0.84		0.09	0.14
WCST	−0.03	−0.08	0.03	0.03	−0.14		
CPT-III	−0.21	−0.40	−0.01	0.10	−0.32 *		
Step 1							
Constant	23.81	21.11	26.51	1.33		0.20 *	0.12 *
WCST	−0.03	−0.09	0.02	0.03	−0.174		
CPT-III	−0.18	−0.37	0.01	0.09	−0.284		
Fixation time (ms)	2.80	0.00	0.00	0.00	0.347 *		

Note. CI: confidence interval; CPT-III: Conners’ Continuous Performance Test-III; LL: lower limit; ms: milliseconds; SE: standard error; UL: upper limit; WCST: Wisconsin Card Sorting Test; R^2^ = adjusted R^2^; ΔR^2^ = change in R^2^. * *p* ≤ 0.05.

## Data Availability

Due to the characteristics of the sample and for data protection reasons, all data supporting the findings of this study are available from the corresponding author upon request. Access to the data is granted solely for academic and research purposes, and all requests will be reviewed to ensure compliance with privacy and ethical considerations.

## Abbreviations

The following abbreviations are used in this manuscript:

AOI	Area of Interest
CI	Confidence Interval
CPT-III	Conners’ Continuous Performance Test-III
IPV	Intimate Partner Violence
LL	Lower Limit
M	Mean
SD	Standard Deviations
TAS-20	Toronto Alexithymia Scale-20
UL	Upper Limit
WCST	Wisconsin Card Sorting Test
